# Effects of Carpal Tunnel Syndrome on Reach-to-Pinch Performance

**DOI:** 10.1371/journal.pone.0092063

**Published:** 2014-03-14

**Authors:** Raviraj Nataraj, Peter J. Evans, William H. Seitz, Zong-Ming Li

**Affiliations:** 1 Department of Biomedical Engineering, Cleveland Clinic, Cleveland, Ohio, United States of America; 2 Department of Orthopaedic Surgery, Cleveland Clinic, Cleveland, Ohio, United States of America; 3 Department of Physical Medicine and Rehabilitation, Cleveland Clinic, Cleveland, Ohio, United States of America; Emory University School Of Medicine, United States of America

## Abstract

**Background:**

Carpal tunnel syndrome (CTS) compromises fine sensorimotor function during activities of daily living. Reach-to-pinch for a small object requires not only dexterity of the grasping digits, but also coordinated transport of the hand to the target. This study examined the effects of CTS on the kinematic performance of reach-to-pinch maneuver.

**Methods:**

Eleven CTS subjects and 11 able-bodied (ABL) controls donned markers for motion capture of the hand, thumb and index finger during reach-to-pinch. Subjects were presented with a virtual target they could see without seeing their reaching upper-extremity. Subjects were instructed to reach to and grasp a virtual object as accurately and precisely as possible. Performance was assessed by variability of the movement trajectories of the digits and hand, the accuracy relative to the target, and precision of pinch contact over repetitive trials.

**Findings:**

The CTS group demonstrated significantly increased movement variability in inter-pad distance, joint angles, and transport of the hand compared to ABL controls (p<0.01). CTS subjects also exhibited reductions in accuracy (41%) and precision (33%) of their pinch contact location (p<0.05).

**Interpretation:**

CTS adversely affects the ability to execute the reach-to-pinch maneuver. Reduced performance was shown in terms of increased variability for both grasp and transport and the ability to locate the grasping digits relative to a target-object. These performance indices could be used for diagnostic and evaluative purposes of CTS.

## Introduction

Reach-to-grasp is a fundamental action in performing activities of daily living. It requires coordinated movements of both the reaching hand and the grasping digits relative to the target object [1]. To grasp smaller objects, the thumb and index finger are utilized to execute precision pinch in conjunction with transport of the reaching hand [2]. Regulation of the reach-to-pinch maneuver by robust sensorimotor feedback is critical to ensure the movement action is executed efficiently and reliably prior to securing grip.

Individuals with carpal tunnel syndrome (CTS) suffer from sensorimotor dysfunction of the hand due to compression of the median nerve at the wrist. With CTS, thumb and index finger function is afflicted by pain emanating from the wrist and palm, general tingling, and motor deficit of the thenar and lumbrical muscles. It has been previously demonstrated that CTS impairs contact variability of the thumb and index finger during precision pinch movement [3]. This observation is largely attributable to the nature of CTS as a peripheral neuropathy directly compromising median nerve innervation to muscles of the thumb and index finger. However, the effects of CTS may be more extensive to the function of the entire upper-extremity as diminished sensorimotor processing associated with CTS can produce disorganization at the central level [4]. While CTS is noted as a peripheral neuropathy, its effects on central-level changes have been demonstrated by altered representations at the primary sensory cortex [5]. Characterizing the underlying effects of CTS may provide further insight into its functional consequences and guide considerations for its diagnosis and treatment [6], [7].

In order to characterize the sensorimotor effects of CTS on hand and upper extremity function, the reach-to-pinch task is well-suited. It involves precision grasp of smaller objects by the thumb and index finger digits in conjunction with transport of the hand. Kinematic dyscoordination is marked by movement variability of the digits and hand due to CTS-associated alterations in peripheral and central-mediated control. Visual feedback processing at the central-level is integral for controlling manual prehension [8]. Furthermore, visual feedback can compensate for sensorimotor dysfunction during reaching movements [9]. Therefore, visual feedback needs to be controlled in order to properly assess the sensorimotor ramifications of CTS on functional movement.

In this study, we examined the effects of CTS on reach-to-pinch function while eliminating the confounding contribution by visual feedback. It was hypothesized that (1) individuals with CTS would exhibit increased movement variability of the index finger and thumb relative to the hand during the reach-to-pinch maneuver compared to able-bodied (ABL) controls. It was additionally hypothesized that (2) CTS would produce more variable hand trajectories. Finally, it was hypothesized that (3) CTS subjects would be more inaccurate and imprecise in localizing their digits relative to the target location. Confirmation of these hypotheses would demonstrate the kinematic effects CTS has not only on the afflicted grasping digits, but on overall upper-extremity function.

## Methods

### Human Subjects

A total of twenty-two subjects (11 ABL, 11 CTS) between the ages of participated in this study. Subjects were age- and gender-matched between the two population groups of ABL and CTS. Each group consisted of 9 females and 2 males with mean age of 49.5 ± 9.6 years for CTS and 48.6 ± 7.6 years for ABL. All subjects were right-hand dominant, as verified by the Edinburgh Handedness Inventory [10]. Subjects recruited into the CTS group were diagnosed according to observations related to the following clinical criteria: 1) history of parathesias, pain, and/or numbness in the median innervated hand territory persisting for at least 3 months; 2) positive provocative maneuvers including Tinel’s sign, Phalen’s test, and/or median nerve compression test; 3) abnormal electrodiagnostic testing consistent with median nerve neuropathy at/or distal to the wrist [11]; 4) an overall CTS Severity Questionnaire [12] score greater than 1.5; (5) positive diagnosis according to clinical discretion [7]. The ABL subjects did not previously report or demonstrate a history of disease, injury, or previous complications involving the hand and upper extremity. CTS and control subjects exclusion criteria included: 1) electrodiagnostic tests, if underwent, indicating ulnar, radial, or proximal median neuropathy; 2) existence of a central nervous system disease (e.g., multiple sclerosis, myasthenia gravis, Parkinson’s disease); 3) pregnancy; 4) history of trauma or surgical intervention to the hand/wrist; 5) rheumatoid arthritis or osteoarthritis of the hand/wrist; 6) diabetes; 7) a steroid injection to the hand within the past 3 weeks. All participants across both groups signed an informed consent approved by the local Institutional Review Board. All CTS subjects were diagnosed as having at least moderate severity of CTS such that surgery was a recommended option. CTS subjects did not demonstrate notable muscle atrophy according to clinical assessment. CTS subjects exhibited pinch strength (53.1±18N) similar to the ABL subject group (57.2±18N).

### Computation of Digit Kinematics

**Figure 1:**
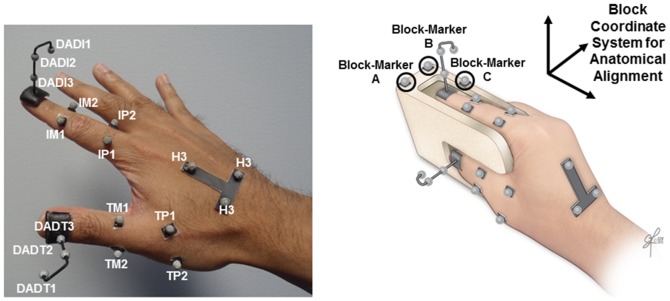
Experimental set-up. LEFT**:** Markers utilized for motion tracking and computing digit kinematics [13] RIGHT: Calibration using a digit alignment device [14].

Retro-reflective markers were affixed to the dorsal surface of the right hand of each subject to derive thumb and index finger kinematics. The 3-D position of each marker was tracked at 100Hz using a motion capture system (Model 460, Vicon Motion Systems and Peak Performance, Inc., Oxford, UK). A marker set established in our laboratory was employed to compute joint kinematics with considerations of anatomical alignment (**Figure 1A**) [13]. To explicitly define the position and orientation of the distal digit segment of the thumb and index finger, the marker set included a nail marker-cluster employed with a digit alignment device (DAD, **Figure 1B**) [14], [15]. The nail-cluster was placed on the dorsal side of the digit with experimenter visual observation that the long-axis of the nail-cluster stem was approximately in-line with the central prominence of the finger-pad.

### Experimental Protocol

**Figure 2:**
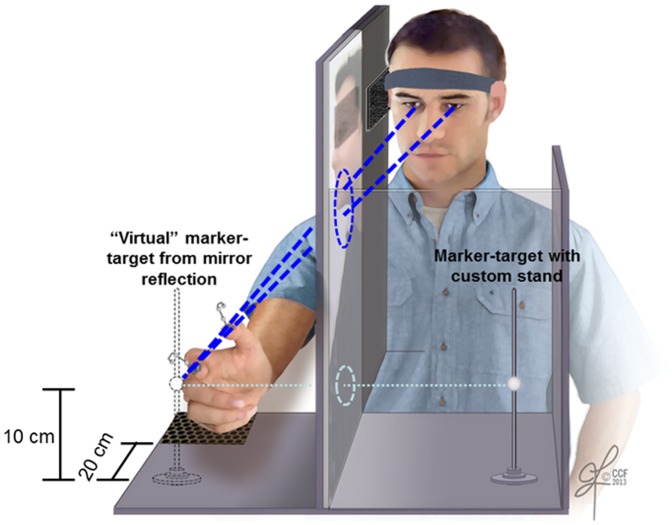
Subject performs reach-to-pinch maneuver towards “virtual” target with no visual feedback of moving hand and grasping digits.

A custom platform-rig (**Figure 2**) was constructed to perform the reach-to-pinch experiments without visual feedback of the reaching hand while allowing visualization of the target to be grasped. The rig consisted of housing with a central slit to accommodate a high-resolution mirror (Imperial Glass & Door Company, Cleveland, OH) maintained in a vertical position. The mirror was approximately 15 inches high, 20 inches deep, and 1/8 inch thick. With the mirror in place, the rig was divided into two alleys with the reflective side of the mirror facing the left alley. The subject was seated facing forward directly in front of the left alley, which contained a marker-target placed on a custom stand. While sitting with head, body and shoulders square to the left alley, the subject was able to clearly gaze the reflection of the marker-target in the mirror. The subject was asked to maintain consistent trunk and head posture across all trials. The subject was instructed to treat the virtual target reflection as if it were a real target that could be physically contacted with the right hand. Since the subject’s reaching right hand was behind the mirror in the right alley, the subject had no visual feedback of the reaching hand. A head-strap with right-side blinder was employed to ensure that the subject received no movement cues from the reaching upper-extremity. The subject was instructed for each reach-to-pinch trial to maintain visual engagement with the reflection target while making the reach maneuver and to pinch the target with the thumb and index finger as accurately and as consistently as possible.

Prior to the commencement of each trial, the experimenter asked the subject to be ‘ready’, whereby the seated subject placed the right hand, ulnar-side down, onto the designated starting area (towards platform corner) of the right alley with the index finger and thumb lightly in contact. The three non-involved digits (middle, ring, and little fingers) were comfortably curled. Since subjects were not able to view the reaching hand, they relied on tactile feedback to replace the hand on the starting area, which had a distinct texture from the rest of the platform. To control for reach-to-pinch maneuver speed across subjects, the subject listened to an audio-metronome producing moderate-pitch beeps at a frequency of 1 Hz throughout the experiment. To commence each trial, the experimenter provided an audible ‘go’ command, after which time, the subject self-selected which beep to commence the reach with the right hand towards the target reflection. The subject paced the maneuver such that pinch contact was made on the next (second) audible beep, and then immediately began to return the hand to the starting position on the third beep to complete the trial. The entire reach-to-pinch-to-return maneuver for each trial was approximately two seconds. A total of 30 consecutive reach-to-pinch trials were executed with approximately a few seconds elapsing between trials according to experimenter discretion.

### Computation of Digit-Pad Contact

Using the nail marker-cluster as a reference for an aligned 3-D coordinate system, a spherical model of the respective digit-pad was represented. A virtual “nail-point” is computed as a projection along the marker-cluster stem to the dorsal surface of the nail and served as the respective sphere “center”. Using digital calipers, the digit thickness was measured as the transverse distance from dorsal surface to digit-pad prominence of the distal segment for both the thumb and index finger and served as the sphere “radius”. The distance between the digit-pad surfaces was subsequently denoted as “inter-pad” distance. The *contact* between the thumb and index finger was estimated to occur when inter-pad distance and inter-pad velocity were below thresholds of 1 mm and 20 mm/sec, respectively.

### Statistical Analysis

Comparisons between the ABL and CTS groups were made using the Mann-Whitney-Wilcoxon non-parametric test for variables. These variables include mean trajectory value and global pinch contact location. A paired t-test was used for mean trial-to-trial variability. Trajectory *variability* was defined as the 1 standard deviation (s.d.) band about the mean trajectory for each subject across 20 equally-spaced points defined for each pinch cycle (i.e., open → closed → open). When comparing between groups, the variability for one group was “normalized” to be on the same scale of the other group since absolute variability generally increases proportionally with range of movement. Thus, the normalization factor multiplying the variability of group B to scale to group A is range(A)/range(B). To consider differences in hand sizes, variables of inter-pad distance and digit path-lengths were normalized by respective subject palm width.

## Results

**Figure 3:**
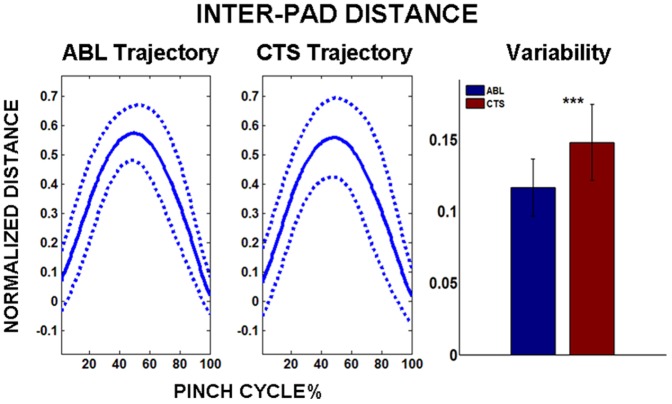
Inter-pad distance. LEFT: Inter-pad trajectory over pinch cycle for ABL and CTS subject groups. Solid line denotes mean trajectory, and dotted line denotes variability (±1 s.d.) about mean trajectory. RIGHT: Comparing variability between ABL and CTS groups. Note: ***p<0.001, Distances self-normalized by subject “palm width”.

**Figure 4:**
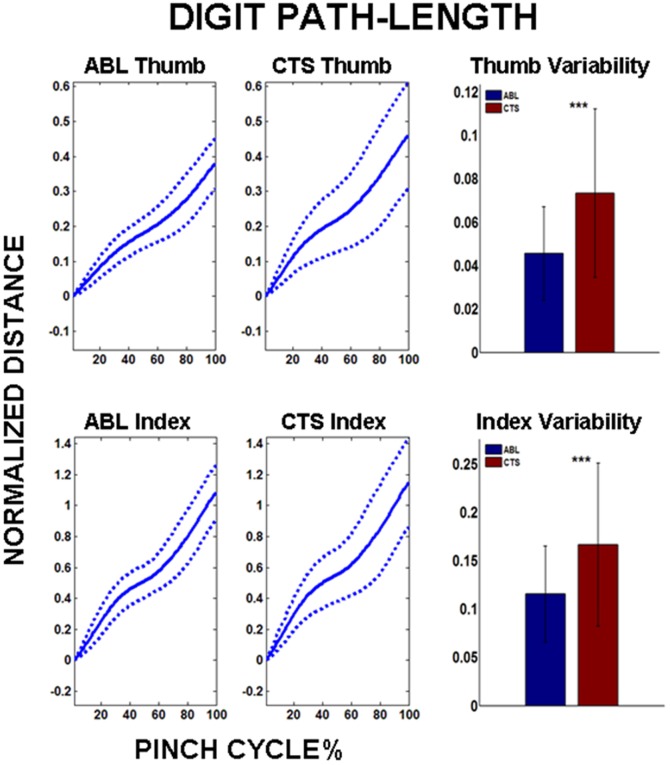
Digit path-length accumulated over pinch cycle. TOP: Mean trajectory and variability of thumb path-length for ABL and CTS groups. BOTTOM: Mean trajectory and variability of index path-length for ABL and CTS groups. Note: ***p<0.001, Distances self-normalized by subject “palm width”.

The inter-pad distance trajectory across the pinch cycle is shown in **Figure 3** for both the ABL and CTS groups. While the mean trajectory is similar across both groups, the variability about the respective mean is significantly larger for CTS (p<0.001). The inter-pad variability increased by 26.5% with CTS subjects in comparison to ABL controls. Correspondingly, the path for the distal segment of each individual digit also demonstrated increased variability for the CTS group. The path variability for both the thumb and index finger was significantly greater (p<0.001) for CTS than ABL (**Figure 4**). The path-length was smaller for the thumb than the index finger for both subject groups, however, no significant differences were observed for path-lengths across subject groups.

**Figure 5:**
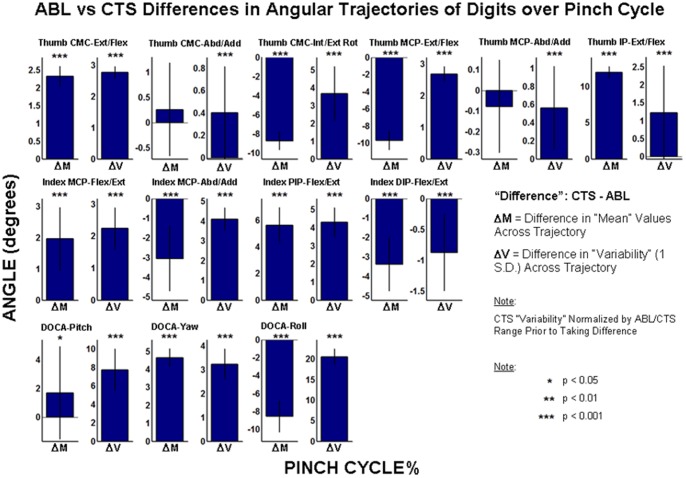
Differences (Δ) in mean-value (M) and variability (V, ±1s.d. about mean trajectory) of mean angular trajectories of digits between ABL and CTS groups.

The differences in the mean and variability values of the angular trajectories of the digits across the pinch cycle are shown in **Figure 5** for the joints of interest and the relative orientation of the distal thumb and index finger segments (i.e., DOCA). The difference in variability about the mean angular trajectory was significantly different from zero for all DOFs (p<0.001). The difference was positive (i.e., CTS variability greater than ABL) for all angular metrics observed except for flexion/extension angle at the index DIP joint. The difference in mean trajectory value was significantly different from zero for 8 out of 10 joint DOFs (p<0.001). Significant non-zero differences between subject groups were observed for all three DOCA angles: DOCA-Pitch (p<0.05), DOCA-Pitch (p<0.001), and DOCA-Roll (p<0.001).

**Figure 6:**
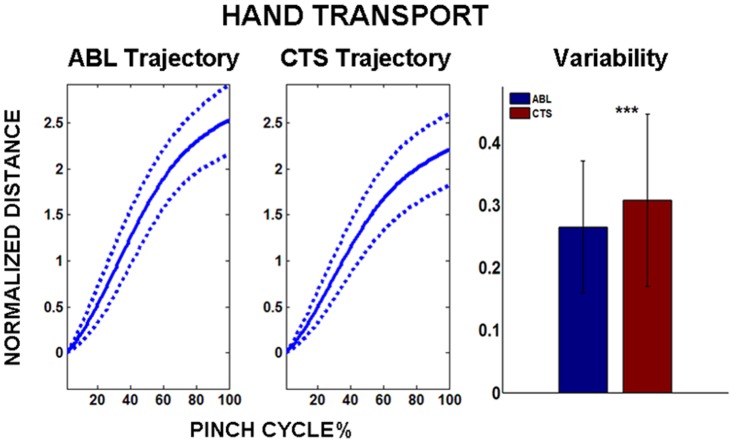
Hand transport accumulated over pinch cycle. LEFT: Hand transport for ABL and CTS subject groups. Solid line denotes mean trajectory, and dotted line denotes variability (±1 s.d.) about mean trajectory. RIGHT: Comparing variability between ABL and CTS groups. Note: ***p<0.001, Distances self-normalized by subject “palm width”.

While inter-pad distance, digit path-length, and digit angular metrics describe pinch regulation at the digit-level, hand transport metrics indicate performance of the reaching component. **Figure 6** shows the mean hand transport accumulated over the pinch cycle for both ABL and CTS groups. The CTS group demonstrated a mean reduction in hand transport of 12.7%, but this reduction was not significant (p>0.05). Compared to ABL, the CTS subject group exhibited a 16.2% increase in variability about the mean trajectory for hand transport. This increase in variability across the pinch cycle was significant (p<0.001).

**Figure 7:**
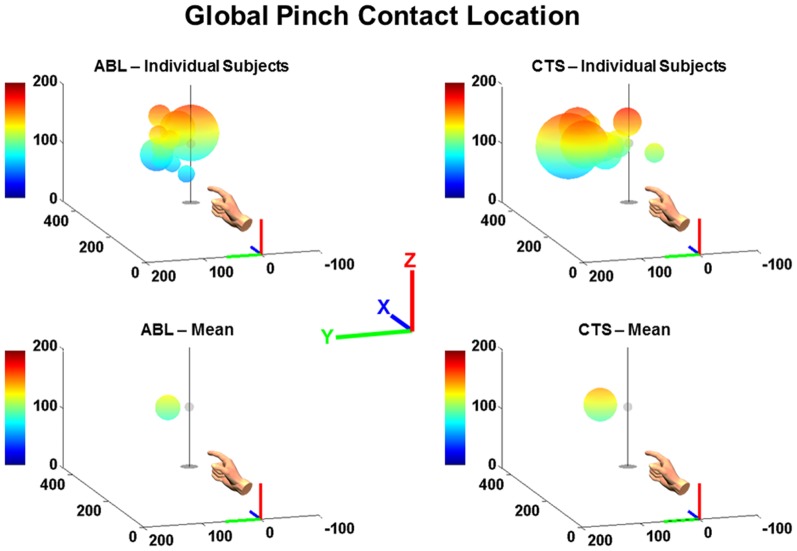
Mean pinch contact location for individual ABL and CTS subjects relative to virtual target, pictured with stand. Sphere center denotes mean pinch contact location and radius denotes pinch precision (1 s.d.) about that location. Pinch contact spheres shown relative to pinch target silhouette with respect to global coordinate system (X  =  +forward, Y  =  +left, Z  =  +up). Note: Color bar denotes height and transparency overlay indicates perceptive depth for 3-D visualization.

The mean locations and variations of pinch contact at termination of the reach-to-pinch maneuver are shown in **Figure 7** for each individual subject and across each subject group. Each pinch contact location relative to the virtual target is symbolized by a pinch sphere whose center and radius denote the mean contact point and the variation (±1 s.d.) about that point, respectively. These pinch contact results are summarized in **Table 1**. The mean pinch contact location for both subject groups was proximal (negative X-coordinate), left (positive Y-coordinate), and above (positive Z-coordinate) the target. The CTS subject group demonstrated a 41% reduction in accuracy relative to the target with a mean pinch error of 80.3 mm compared to 56.8 mm for ABL subjects. The CTS subject group also demonstrated a 33% reduction in precision, with variation (1 s.d.) about their mean contact location of 27.6 mm, compared to 20.8 mm for the ABL group. The reductions in accuracy and precision with CTS compared to ABL were significant (p<0.05).

**Table 1:** Global Pinch Contact Location Metrics.

	Mean Pinch Location from Target (X, mm)	Mean Pinch Location from Target (Y, mm)	Mean Pinch Location from Target (Z, mm)	Mean Pinch Error (mm)	Mean Pinch Precision (mm)
ABL	−7.6	40.0	3.3	56.8	20.8
CTS	−30.2	56.3	14.4	80.3	27.6
Δ(CTS-ABL)	−22.6	16.3	11.1	23.5*	6.8*

Note: *significant difference at p<0.05; +X  =  forward, +Y  =  leftward, +Z  =  upward relative to target.

## Discussion

In this study, the effects of CTS on the kinematics of both the reaching and grasping components for the reach-to-pinch maneuver were investigated. Compared to the ABL group, the kinematic variability of the pinching digits and in transporting the hand were increased with CTS. The ability to accurately and precisely locate the grasping digits onto the intended target also was found to be diminished with CTS. Ultimately, these deficits in kinematic performance in the presence of CTS suggest sensorimotor impairment, which may explain the functional clumsiness classically reported with CTS [16].

Sensorimotor dysfunction associated with compression of the median nerve would produce negative focal effects in controlling motion of the thumb and index finger relative to the hand. The muscles being innervated by the median nerve include the first lumbrical and those in the thenar group. The first lumbrical inserts into the radial side and onto the extensor expansion of the index MCP joint. With the MCP joint being the most proximal in the kinematic chain of the index finger digit, dysfunction at this joint would explain the sensitivity to CTS upon the variability in the path-length of the distal portion of the index finger and consequently related effects on inter-pad distance. The median nerve innervates the thenar muscles which produces abduction, flexion, and opposition function of the thumb. Therefore, CTS effects to increase variability of distal thumb path-length were expectantly observed.

While increases in variability were evident, differences in the range of thumb and index path-length and inter-pad distance between CTS and ABL subject groups were not found to be significant. It was plausible to expect decreases in ranges of movement with CTS, since the symptom of pain, typically associated with CTS, can produce functional disincentive to approach the range of movement extrema [17]. Furthermore, chronic CTS can reduce range of motion because of increased joint rigidity due to changes in passive tissue structures. However, the mean range of inter-pad distance and digit path-lengths for the CTS group were likely found to be similar to the ABL group in this study since the reach-to-pinch function does not require large changes in digit pinch span to grasp smaller objects [18].

The increases in variability due to CTS were not only observed for linear kinematic parameters but also for parameters describing angular variations at the digit joints and the distal orientation of the grasping digits. The variability for nearly all observed angular parameters across joints of both digits were found to be greater for CTS. The increase in CTS variability for DOCA-Yaw and DOCA-Roll may be specifically due to the lowered ability to consistently bring the thumb in opposition. There were also significant differences in the mean trajectory value for several joint DOFs between CTS and ABL. Regardless of these differences being positive or negative, significant angular offsets indicate that CTS subjects generally assumed a pinching posture that was distinct compared to that of the ABL group. Overall, these changes in controlling digit orientations may indicate the sensorimotor and proprioceptive deficits produced by CTS.

Although CTS is generally classified as a peripheral neuropathy, the functional consequences can go beyond that of the grasping digits with articulating muscles being directly innervated by the median nerve. Results from this study demonstrate that there is increased variability in transport of the reaching hand with CTS. Transport of the reaching hand depends on coordinated movement the proximal joints of the upper extremity including the elbow and shoulder. CTS may produce functional impairment at these joints by a multi-faceted mechanism. Evidence exists that control of the reaching and grasping components may be closely coupled [2]. Therefore pain, tingling, and numbness elicited by CTS in the grasping hand may produce a compound effect that impairs composite sensory integration and motor output to consequently alter the entire reaching movement pattern. Furthermore, it has been shown that chronic CTS is associated with long-term, adaptive changes in the central nervous system as indicated by modified cortical representations [5]. These changes in central-level muscle control may yield maladaptations that adversely affect movement trajectory of transport in addition to grasp.

While CTS appears to promote increased variability in movement trajectories associated with the reach-to-pinch maneuver, functional task performance may be best characterized at maneuver termination. Ultimately, reliable contact with the target-object is the end-goal of the maneuver itself. While transport largely brings the hand within the vicinity of the target, pinch contact upon the target is not completed until the thumb and index finger are brought together to enclose upon the target. Both components contribute to global localization of the pinch contact point. Therefore, comprehensive performance across both the reaching and grasping components of the reach-to-pinch maneuver can be assessed by accuracy and precision of the mean contact location observed relative to the virtual target. It was observed that the CTS group performed the task with reduced accuracy and precision compared to the ABL control group.

On average, the ABL group was able to locate the digits as close as 57 mm to the virtual target upon pinch contact, while the CTS subjects exhibited a mean accuracy that was 41% worse than ABL. Even for ABL subjects, visual feedback of the reaching hand is necessary to improve accuracy in localizing the grasping digits relative to a target object as part of planning and execution of goal-directed movements [19]. Both subject groups demonstrated a mean offset in pinch contact that is behind, left, and above the target with the CTS bias being greater than ABL in each dimension. Furthermore, it has been suggested that when grasping a virtual object without representation of the reaching hand, the disparity between hand and object is a necessary cue in conjunction with binocular vision for accurate grasp localization [20]. In this study, the *increased* error relative to the target for the CTS group, particularly in the forward-backward dimension may be due to CTS-associated sensory dysfunction, including pain which may disincentivize further reach. Assuming experimental biases to be consistent, then precision of pinch contact upon the perceived target location should be more independent of bias effects, visual or otherwise. Therefore, the reduced precision observed for CTS subjects relative to the ABL group may be mainly attributable to sensorimotor dysfunction.

The ramifications of carpal tunnel syndrome on upper-extremity function may include effects from both reduced motor output and compromised sensory feedback. Previous studies have observed the actions of visual feedback on reach-to-grasp function to illuminate whether grasp and transport may be mediated by distinctly separate modes of control [21] or belong to a more integrated control scheme [22]. Visual sensory feedback has also been controlled for determining the relative contribution of vision and proprioception to normative execution of the task [23]. It has also been shown that vision can be employed to compensate for compromised proprioceptive feedback for individuals with movement pathology [9]. Therefore, it was critical that vision of the reaching upper-extremity was removed to focally attribute the effects of CTS upon subsequent functional performance. It is possible that CTS subject would heavily rely on visual feedback of the hand and arm to compensate for reach-to-pinch performance. In the future, it would be of interest to examine to what degree individuals with CTS do specifically rely on visual feedback as a compensatory mechanism in performing reaching tasks.

Ultimately, the kinematic dyscoordination observed in this study may be attributable to several facets associated with carpal tunnel syndrome. Certainly, the sensorimotor dysfunction from the peripheral neuropathy produced from compression of the median nerve is likely to play a role. Furthermore, long-term effects due to chronic pain or central-level adaptations may have fundamentally altered motor patterns for individuals with CTS. Regardless of the exact mechanism of kinematic dysfunction, this study demonstrates that CTS has negative consequences throughout composite movement functions of the hand and upper extremity as both grasp and transport are adversely affected. Future work may be performed to better identify the underlying mechanism of CTS by determining whether resulting performance is more sensitive to CTS-associated changes at the peripheral- versus the central-level of nervous system processing. In any case, the reach-to-pinch paradigm presented in this study may provide a highly prevalent basis to assess the severity and specific nature of CTS on a functional level. Given that no standard clinical test has clearly demonstrated the highest sensitivity or specificity in diagnosing CTS for subsequent treatment [7], advanced methods for reliably testing the outcome effects of CTS and its intervention are continually developed [24]. To comprehensively observe the effects of CTS as it relates to complex activities of daily living, functional tests involving more tasks more closely related to those of ADL are needed.
